# Book Reviews

**Published:** 1819-05

**Authors:** 


### CRITICAL ANALYSIS

### OF RECENT PUBLICATIONS,

IN THE

### DIFFERENT BRANCHES OF MEDICINE, SURGERY, &c.

*Transactions of the Association of Fellows and Licentiates of the King's and Queen's College of Physicians in Ireland.* Vol. II.—8vo. pp. 602.

*(Continued from p. 323.)*

### VII.—*An Account of the Extirpation of a Tumor of the Neck, engaging the Parotid Gland*; by RICHARD CARMICHAEL, M.R.I.A.

THIS is the history of a case, of which such an abstract as our limits will admit of would convey but little useful information. We shall, therefore, merely observe, that it was a sarcomatous tumor, of firm structure, situate behind the ear and angle of the lower jaw, nearly five inches in diameter, engaging the parotid gland, and attached to the basis of the skull between the mastoid and styloid processes, and to the transverse process of the first cervical vertebra; that was removed by an operation, the difficulties attendant on which were only exceeded by the dexterity with which it was executed. There are two indications of considerable practical importance furnished by this case:—1, that, in removing tumors situate like that to which we allude, a ligature should be passed under the trunk of the carotid artery in the first instance, to be tightened if requisite; for, from placing reliance on the powers of the fingers of an assistant for the compression of the artery in this case, the patient was in imminent danger of losing his life from hæmorrhage;—2, that low delirium, with capability to reply correctly to short questions distinctly proposed, accompanied with quick and weak pulse, inquietude, and a disposition to be rising out of bed, coming on after long and painful operations, may be promptly relieved by wine and opiates. These are amongst the reflections deduced by Mr. Carmichael from the history of this case.

### VIII.—*A Case on the Use of Turpentine;* by WHITTOCK NICHOLL, M.D. F.L.S. &c.

This was a case of melæna, apparently arising from irritation and diseased secretion from the mucous membrane of the intestinal canal, affecting a girl twelve years of age. The turpentine was exhibited after the black colour of the stools had disappeared, but when morbid irritation still seems to have existed. One drachm and a half of oil of turpentine, in six drachms of syrup of tolu, was given as a dose; two hours after the same was injected as a glyster, and half an ounce of castor oil given by the mouth. The symptoms "vanished as by the force of magic, and the patient rapidly recovered her health."

IX.—*A Case of Angina Pectoris;* by the same.

This case terminated fatally, after having existed about two months; but, from the want of examination after death, it is not calculated either to excite much interest or to contribute to our knowledge respecting the nature of that affection.

The same paper contains an account of a case of apparently chronic inflammation of the heart, in a man, aged about 30. James's powder, ipecacuanha, and calomel, were exhibited for a few days, and blood-letting was once employed, without much evident benefit; when a seton was made over the region of the heart; a grain of opium and digitalis were also directed for him, to be repeated after intervals of a few hours. The patient, the next day, said, that

"From the moment of the insertion of the seton he entirely lost the pain in the heart; that the palpitation had scarcely returned, and the medicine procured sleep and quieted him. Strict and entire quiet of body and mind, with abstinence from all nourishing or stimulating diet, were rigidly adhered to.

"October 7.—No symptom, but debility.

"Nov. 10.—Seton removed.

"12th.—Had cold sweats since the removal of the seton, which disappeared after his having recourse to a small dose of salts repeated daily. His pulse maintained, when I last saw him, which was in the middle of November, its full prominent character; and, although he called himself well, he was nevertheless unfit, in my opinion, for any situation which called for any degree of exertion. I have just heard (February 8, 1818,) that he has been tolerably well ever since I saw him, but that he is subject to returns of palpitation."

X.—*Case of Sarcoma or Polypus in the Colon;* by EDWARD PERCIVAL, M.B. M.R.I.A. &c.

The subject of this case, a child, aged twenty-one months, died in consequence of constipation of the bowels, which had been coming on gradually for about five weeks.

"Some livid patches were found on the colon, and some extent of inflammatory appearance on its villous coat, about the middle of the intestine. Here the gut adhered on all sides to a firm tumor, about the size of a hazel-nut, forming a complete cul-de-sac. On separating those parts which adhered by recent inflammation, an excrescence was discovered, of glandular texture, deeply seated in the parieties of the intestine. Its external hue was dark or livid; its internal colour bluish grey. At the base of this tumor a small cavity and ulcer appeared, which had nearly eroded the tunics of the gut.

"The rectum was contracted in its capacity, empty and clean.

"Compare Morgagni Ep. xxxi. 21; Portal Anat. Med. tome v. p. 243; Monro's Morbid Anatomy of the Gullet and Stomach, p. 190; Baillie's Morbid Anat. p. 193."

### XI.—*Two Cases of Inflammation and Enlargement of the Pancreas;* by the same.

This is a paper of considerable value; for, although the disease of which it treats is not of frequent occurrence, yet, as Dr. Percival remarks, the difficulty of ascertaining the condition of this organ by any other symptoms than such as are secondarily manifested in the stomach or liver, must render our opinion on many cases uncertain, and our practice erroneous.

"When pain and tumor of the pancreas (says the author) are mistaken for inflammation of certain parts of the liver, the remedies of depletion, which are applicable to both, may resolve the disorder, before the error of diagnosis is discovered. But a more obvious and fatal error may arise from confounding the pain and jaundice caused by the pressure of an inflamed pancreas upon the ductus communis choledochus, with spasm or calculous obstruction of that canal; and it is chiefly with a view to obviate this mistake, and its consequences, that I offer the following cases to the notice of the Association."

In one of the cases that occurred to the observation of Dr. Percival, which terminated fatally,

"On examining the abdomen, the pancreas, greatly enlarged, was found occupying the place of the tumor before felt in the epigastrium. The ductus communis choledochus was imperforate in the parts adjacent to the pancreas, and where its pressure had been greatest. The gall-bladder was full, and the cystic duct pervious; the substance of the pancreas was scirrhous, and, on being cut into, was found to contain a considerable abscess. The kidneys were sound, but the liver was much diseased.

"A case of scirrhous pancreas, which illustrates some points in the foregoing histories, is related by Mr. Todd in the first volume of the Dublin Hospital Reports, &c. art. xiv. The reader is also referred to a paper by Dr. Latham, in the 11th volume of Transactions of the College of Physicians, art. v. which contains some very just observations on the diagnosis of abdomical tumors."

We shall transcribe the account of the case which terminated favourably, as it will point out the diagnosis of this disease, and the medicinal measures likely to be most beneficial.

"An elderly gentleman, inclining to corpulency, was frequently seized, in the last autumn, with acute pains in the epigastrium, attended with soreness and tumor of the parts, and followed by jaundice. His medical attendant prescribed cordial draughts and mercurial purgatives, which relieved, but did not remove, the urgent symptoms. After some weeks, the patient began to experience an obscure pain in his chest on making any bodily exertion; but it was unattended with cough or any difficulty or disturbance of the respiration. As this pain or uneasiness appeared to be associated with the distress in the epigastrium, a blistering plaster was applied to the latter part with much benefit: neither local nor general blood-letting had been resorted to.

"On the 10th of November I saw this gentleman, two days after his arrival in Bath. His complexion was dingy, and yellow about the eyes; his tongue much furred; his urine scanty and high-coloured; his pulse, in the morning, did not exceed 78 strokes in the minute. On viewing the epigastrium, I found it prominent, especially in the central part, between the umbilicus and scrobiculus cordis. The verge of the liver was pretty clearly ascertainable, bearing free pressure, and betraying no sign of organic disease; but detached from this organ, in the gastric region, was an unusually dense mass, tender to the touch, and yielding slowly on pressure. The gentleman complained of chilliness in an evening, and his flesh and strength were much wasted. Eight leeches applied to the epigastrium bled copiously, and produced some faintness. The tumor and tenderness were considerably reduced; the urine became free and of natural appearance, the pulse fuller and softer; and the patient expressed himself greatly relieved from his oppression. A vesication on the same parts, kept open for ten days, with the use of saline aperients, an occasional warm bath, and a restricted diet, so far restored the patient, that he assured me he had not, for a long time, felt himself in such good general health. Still a tumor and hardness were discernible in the epigastrium; until a spontaneous diarrhœa occurring, it shrunk rapidly in its dimensions, and before the expiration of a fortnight (Dec. 8th) entirely disappeared.

"It is deserving of remark, that, in the progress of this subsidence, the tumor assumed a more distinct form, like the smaller extremity of an egg, and preserved this shape till it could no longer be felt.

"Some slight vestiges remained of the obscure pain or uneasiness in the chest on making any unusual bodily exertion, or sometimes even in walking at a gentle pace; but neither the pulse nor the respiration were, in the slightest degree, affected under these circumstances."

Dr. Crampton also adduces two cases of a similar disease; one of which terminated favourably, by the use of measures similar to those employed by Dr. Percival; the other ended fatally. The morbid appearances were analogous with those related by Dr. Percival.

In the 25th volume of our Journal, an instance is given of death ensuing from rupture of the gall-bladder, consequent on the obstruction of the ductus communis choledochus from an enlarged and indurated pancreas.

XII.—*Clinical Report on Dropsies;* by John Crampton, M.D. Honorary Fellow of the King's and Queen's College of Physicians, &c. &c.

This Memoir contains an extensive series of facts of the most interesting nature in themselves, and of which the value is much increased by the clinical reflections of Dr. Crampton, which are deduced from very accurate observation and the most correct and enlightened physiological views.

The cases related by Dr. Crampton are seventy-five in number, being all that were admitted into Steevens' Hospital during one year; a correct history of a certain number of cases occurring within a given time, without regard to choice, being best calculated to add to our knowledge respecting the most frequent character of that form of disease.

This Memoir of Dr. Crampton will not, it must be evident, admit of a general abstract adapted to the limits of our Journal: to derive from it all the benefit it is calculated to effect, it must be studied in detail. We shall, therefore, only cite a few of the most striking facts; but those, we trust, will be such as are calculated to excite reflection, and furnish useful information respecting the nature of dropsical affections in general. We shall select them from such cases as terminated fatally, and of which histories of dissection after death are given; as it is those that are best calculated to lead to correct pathological knowledge, and, when this is acquired, the appropriate medicinal measures are sufficiently obvious.

"Case 1.—Mary Callaghan, æt. 22, June 27th, 1817, was admitted with symptoms of very general anasarca: the face, the trunk of the body, the thorax, especially the legs and thighs, were unusually œdematous; the abdomen much distended, and fluctuation evident; pulse small and oppressed, respiration hurried and laborious; urine of a high colour. She suffered excessive pain in the scrobiculus cordis. Dropsical symptoms were present only six weeks.

"She had been given mercurials inwardly by her husband, in the form of solution: this was followed by severe pains in her bowels, the catamenia became suppressed; the swellings then appeared. She attributed her illness to the mercurial medicine.

"On her admission to the hospital, venæsection, to the extent of ten ounces, was twice performed at the interval of a few days: this afforded temporary relief to the pain; the anasarcous swellings left the upper extremities, and there was a considerable diminution in the size of the abdomen. Symptoms, however, of distress in her breathing, return of pain in the scrobiculus cordis, greenish vomiting and hiccup came on; and she died in agony on the 8th of July.

"*Dissection, 9th July.*—Lower extremities anasarcous; only two quarts of fluid in the abdomen; abdominal viscera sound; lungs quite healthy, but adhering in many points; above half a pint of serous fluid, with flakes of lymph, in the pericardium; a white spot on the right ventricle, half an inch in diameter, evidently from a deposition of coagulable lymph."

"CASE 2.—Edward Anderson, æt. 19, July 4, 1817. Anasarca of the face, head, and arms, legs, thighs, and body, has come on in the order of the parts enumerated. Abdomen distended, and contains a fluid; lips and cheeks appear purple and livid; cough, and pain of the left side; pulse small and indistinct, not frequent; urine scanty and red; diarrhœa. Has been dropsical for seven months: it came on immediately after a fall from a car.

"He was partially relieved by a single venæsection, the cough and pain of his side having been removed; in other respects the treatment was ineffectual. He died on the 18th.

"*Dissection, 19th July.*—Body anasarcous; not much fluid in the cavity of the abdomen; liver enlarged, presenting a peculiar marbled appearance; some effusion of lymph about the spleen. The other abdominal viscera healthy; lungs sound, slightly adherent; the heart adhered to the pericardium in many points,—the bands of adhesion appeared not to have been of recent formation."

"CASE 9.—Laurence Mulhall, æt. 45, Oct. 10, 1817. Abdomen very large, tense, and containing a fluid; legs œdematous above ten months; cough and pain of sternum, with frequent pulse; urine red and scanty. Was injured in his chest by falling, and being bruised in a mill: to this accident, in which he was much exposed to wet and cold, he attributed his illness.

"He was directed venæsection to ten ounces on his admission; and a repetition of the same measures on the 13th, as he still complained of the severity of the pain in his chest.

"The brachial artery was punctured in the operation of bleeding, and an aneurism became formed. On the 24th, the whole arm was enormously swelled and blackish; his dropsical symptoms had nearly disappeared.

"He was removed to a surgical ward, where he died on the 20th of November.

"*Dissection, 21st November.*—Thoracic viscera were healthy. Abdomen contained about four quarts of fluid, of a pale straw colour, mixed with flakes of lymph; the liver, spleen, and kidneys, were healthy; but the stomach was one schirrous mass, except a small portion of the cardiac orifice. Omentum could not be discovered, except a small portion which partook of the disease of the stomach.

"The small intestines were much thicker in their coats than natural, and covered externally with small white elevated spots.

"The puncture in the artery was closed up, and the aneurismal pulsation had ceased five days before his death."

"Case 10.—William Tuite, æt. 24, a newsman, November 10, 1817. A relapse of general dropsy, both anasarca and ascites, to a considerable extent; breathing much distressed, pulse hurried, urine scanty and red; no cough or local pain. He was cured of dropsy in September, but, returning to his intemperate habits, and being constantly exposed to cold and wet, the disease recurred.

"He died on the 19th of December, having suffered severe pain in the umbilical region, and excessive distention, for some days antecedent to his death. He would not submit to the operation of the paracentecis.

"*Dissection, 20th December.*—Abdomen was enormously distended with fluid. On opening it, a portion of the omentum was found adhering, by recent exudations, to the umbilicus. Liver diminished in size, but tuberculated throughout: the peritoneal membranes of it were coated with lymph, as well as those of the parietes of the abdomen. Stomach healthy, intestines distended with air; coats of the small intestines were thicker than natural, with effusion between their laminæ. Spleen somewhat enlarged; kidneys healthy. Thoracic viscera in a sound state."

"Case 14.—John Shawe, æt. 60, November 14th, 1817. A very intemperate man, who procured bodies for dissection, had repeated returns of ascites within the last two years: they were generally removed by purgatives, by jalap in prticular, according to his account.

"His first dropsical attack came on with cough and hoarseness. His last illness was present six months: it was preceded by a paralytic attack, during which he lost his speech; from this state he was restored by arteriotomy. His abdomen is now very large, and general anasarca prevails; breathing oppressed; pulse moderate; bowels costive, urine red. A syphilitic eruption is likewise observable all over his body.

"A single venæsection was practised with a view to relieve his breathing; purgatives and mercurials were directed. He died, however, on the 21st November, in an attack of apoplexy.

"*Dissection, 22nd November.*—Abdomen contained a considerable quantity of fluid; liver hard, tuberculated, but very much shrunk in dimensions. Peritoneal covering of the liver coated with lymph. Vessels on the arachnoid and pia mater were turgid and a slight effusion of serous fluid had taken place in the ventricles of the brain.

"The thoracic viscera were healthy."

"Case 15.—John Murtagh, æt. 29, a butcher, April 3, 1818. Sallow and emaciated; much addicted to drinking ardent spirits; enormous œdematous swellings of the lower extremities for twelve months; ascites had supervened within the last three weeks; cough and soreness, with flying pains in his chest and abdomen; pulse frequent, small and hard; urine scanty and high-coloured: attributed his illness to cold.

"Venæsection was directed: it was not practised, as a profuse nasal hæmorrhage came on after the visit. He was slightly relieved by the paracentesis, which was performed on the 9th; but he died on the 16th of April.

"*Dissection*, 17*th April*.—Abdomen was full of a yellowish serous fluid. Liver tuberculated throughout; some of the mesenteric glands were enlarged and indurated. Peritoneum lining the parieties of the abdomen thickened and very vascular, about the iliac region especially; reflections of that membrane over the stomach and intestines much thickened, as well as their muscular coats, seemingly by the intermediate deposition of lymph, as well as a serous fluid."

Histories of thirty-five cases which terminated favourably then ensue, which lead to the following reflections:—

"From the result of the cases recited, it appears that a greater number of dropsies connected with disease of the thoracic viscera were relieved by medicines, and admitted of cure, than those combined with disorders in the viscera of the abdomen. This may, perhaps, appear strange when the vital importance of the viscera of the thorax is considered, and when the opinions of others on this subject are consulted. Ten of the fifteen patients examined after death had either the liver, stomach, or spleen, tuberculated: if any reliance is, therefore, to be placed in a conclusion drawn from so limited a number, ascites, with scirrhous liver, should be considered a more incurable or fatal form of disease even than hydrothorax combined with some organic disorder in the cavity of the chest, provided the organic derangement is at all compatible with the functions of circulation and respiration.

"Of the patients cured, a considerably greater portion were affected with disease in the thoracic viscera; some of them had evidently organic affections of the heart, and yet they appeared to be acted upon by remedies with infinitely more ease than those where disease had established itself in the cavity of the abdomen.

"We should, therefore, not be too confident in our expectations of recovery in ascites, even though the strength be unimpaired, the respiration and the pulse good; nor, on the other hand, should we despair, where the pulse is feeble and intermitting in hydrothorax and the breathing difficult and laborious.

"The diagnosis to ascertain the organ which has been first affected, and which is chiefly oppressed, is extremely desirable, with a view to the mode of treatment and the remedies to be selected.

"In either general or partial dropsy, the preceding cases warrant us in stating that, whenever the organs of respiration appear to labour, if the strength is not much impaired, and if the disease is recent, it will be safe to practise general bleeding: still more so, if, in addition, there are symptoms which denote inflammation of any texture in the cavity of the thorax. In some of the cases, a single venæsection appeared to arrest the progress of a recent dropsical disease; in others, a repetition of that practice seemed necessary to ensure success. In such a complication, other remedies appeared to be thrown away; diuretics would not act, and purgatives did not afford any relief, until after venæsection had been practised.

"After the removal of congestion or of inflammation, should either be present, it is less difficult to regulate the secretions; and, perhaps, there is less nicety in the selection of remedies than is commonly imagined. Blisters, after one or two bleedings, afford relief, on the same principle and in the same manner they do in the other pneumonic disorders not complicated with dropsy.

"If a chronic or a sub-acute inflammatory condition of the viscera in the thorax should maintain a dropsical disease, masked by debility, and not developing itself by its legitimate symptoms, a single bleeding will often tell the true state of the patient, by showing the quality of the blood. In incipient dropsy it is generally buffed, but not always so: at all events, a small venæsection, cautiously practised, can do no harm. The strength of the patient, the state of the pulse and respiration, with the presence or absence of local distress, appear to be better foundations to determine whether venæsection should be practised, than the characters of the urine."

After some observations tending to appreciate the value of the different medicines usually employed in dropsy, Dr. Crampton concludes with remarking that,

"So far as can be collected from the preceding histories, a selection of the diuretics to be employed appears a matter of less consequence than might have been expected: where the medical treatment was directed to prevent or remove those tendencies to organic changes in structure which have been observed to precede dropsical effusion, little then was left for the officinal diuretics to accomplish.

"The plan of treatment where early venæsection in dropsical diseases is recommended, must appear very abhorrent to those who were accustomed to consider the dropsical or serous diathesis as the result of atony or weakness. Relaxation* in the exhalant system is considered one of the general causes of dropsy, according to Dr. Cullen, and blood-letting† one of those practical measures which often gives rise to this relaxed state. Whereas, those who look to the diseased appearances in the different cavities are more disposed to conclude dropsy as associated with an excited condition of the

* Cullen, First Lines, MDCLVI. † Ib. MDCLX.

exhalants pressed by the vis á tergo of the capillaries, and oozing out their fluids more especially on the serous membranes, which are so constructed as not to allow the same distension of their vessels which other textures permit.

"The name of dropsy, and the notions of debility and relaxation, have long tied up the hands of practitioners: it is time that these delusive theories should give place to facts and experiments, and to a reasoning founded on them. It would be well, therefore, in forming our plans of treatment, to lose sight of the name of dropsy, and take measures to prevent those organic changes which we are apprehensive are going on. Nosology, in giving systematic names to diseases, has facilitated the study of medicine; but it inclines us to dwell too much on symptoms, and too little on the real pathological state.

These researches were undertaken with a view also to ascertain the correctness and degree of importance of the statement, or proposition, of Dr. Blackall, that coagulable urine accompanies those dropsies which depend on inflammatory action: the results of the experiments of Dr. Crampton on this point were by no means favourable to these notions.

XIII.—*Cases and Dissections illustrative of Disease of the Brain;* by Samuel Black, M.D. M.R.I.A.

"I am not aware (says Dr. Black) that the following recordsof diseases and dissections of the brain will communicate any thing very new or very uncommon; but I consider it as a principle admirably good in itself, and in its application promotive of the interests of science and of humanity, that every professional man should contribute his quota towards a general fund, from which we may expect to derive accurate histories of disease, and faithful reports of diseased appearances after death."

These cases are apparently described with much perspicuity and accuracy of observation, and the history of them will furnish an addition of some value to those of the various injuries and idiopathic diseases of the brain and the membranes of the cranium, of which the importance has been particularly shown by Pott, Mr. John Bell, Sir Everard Home,* and Dr. Philip Crampton.†

XIV.—*Case of Inflammation and Abscess of the Brain, attended with Disease of the Ear;* by John O'Brien, M.D. &c.

This case is given by Dr. O'Brien as a useful addition to those related by Dr. Black; the observations we made respecting those will equally apply to it.

* *Medico-Chirurgical Transactions*, vol. iii.
† *Dublin Hospital Reports*, vol. i.

XV.—*A Case of Inflammation of the Ear, attended with Symptoms of Compression of the Brain;* by RICHARD GRATTAN, M.D. Fellow and Censor of the King's and Queen's College of Physicians, &c.

This was a case somewhat analogous with the preceding: it occurred in a lady who had been "at all times extremely subject to inflammations of the face and throat, from any incautious exposure to cold." On one occasion of this kind she became affected with acute pain of the ear, which continued to increase for several days, until it was accompanied with symptoms of severe inflammation within the cranium. We cannot enter into a particular history of the symptoms which appeared, and the remedial measures that were had recourse to, but must observe, in a general way, that, under the use of blood-letting, purgatives, saline diaphoretics, calomel, and digitalis, this case terminated in a favourable manner.

Dr. Grattan takes this occasion to adduce some reflections on the nature of inflammation, and on the effects of digitalis and mercury as auxiliaries to blood-letting in the treatment of inflammatory diseases.

We gave a detail of the hypothesis of inflammation adopted by Dr. Grattan, in our last "Historical Sketch of the Progress of Medical Science," not, as we should then have remarked, on account of its novelty, but from its being a clear and lucid display of one that has had several ingenious physiologists for its supporters. This hypothesis was advanced, with nearly all the arguments in favour of it that have since been proposed, by Sig. VACCA, as early as 1765;* and some persons may, indeed, be disposed to consider it merely as a revival of that of ERASISTRATUS.

The hypothesis adopted by Dr. WILSON PHILIP, and which he has endeavoured to substantiate by experiments, is somewhat analogous with this, he says—"Inflammation seems to consist in the debility of the capillaries, followed by an increased action of the larger arteries; and is terminated by resolution, when the capillaries are so far excited, and the larger arteries so far weakened, by the preternatural action, that the power of the capillaries is again in due proportion with the *vis a tergo*."†

The limits of a review will not permit us to enter into a

---

* *Liber de Inflammationis Morbosæ, quæ in humano corpore fit, naturâ, causis, effectibus, et curatione.* 8vo. Fiorenza, 1765.

† *Experimental Enquiry into the Laws of the Vital Functions.* Second edition, p. 283.

full examination of the degree of probability of this hypothesis, and of the experiments on which it was founded: indeed, we consider that the questions which Dr. Philip has himself advanced, as difficulties in the way of it, forcibly show its want of conformity with the general known laws of the animal economy, and thus constitute in themselves the most powerful of theoretical objections. "Why does a failure of power," says Dr. Philip, "of small extent in the capillaries of a vital part strongly excite not only the larger arteries of the part affected, but those of the whole system; while a more extensive debility of the capillaries of an external part excites less increased action in the larger arteries of that part, and often none at all in those of the system in general? Why does inflammation often move suddenly from one part to another, when we see no cause either increasing the action of the capillaries of the inflamed part, or weakening those of the part now affected? Why does inflammation often arise in parts only sympathetically affected, and consequently far removed from the offending cause? Why is inflammation often as apt to spread to neighbouring parts, between which and the part first affected there is no direct communication of vessels, as to parts in continuation with that part?"*

The experiments† of Dr. Philip may be rationally considered as but a weak foundation for such an hypothesis, particularly when we reflect on the circumstances under which they were executed, and the state of the system under which inflammation is most readily excited; its apparent remote causes, and arguments from analogy with what appears to be best substantiated respecting the laws of the functions of the animal economy, all militate against this hypothesis. Several of the above objections will also apply to the hypothesis adopted by Dr. Grattan; and, although this may seem more rational, it is controverted by observation and experiments, not made under such objectionable circumstances as those of Dr. Philip; and it rests on a basis that is merely imaginary, and not supported by any analogous facts. The inflamed sclerotic membrane of the eye is a fair and evident subject for observation, and for thence deducing conclusions respecting the nature of inflammation. The microscope does not here show any increased action of the arteries running to and through the inflamed part. Increased action of the arteries going to an inflamed part seems to be a mere casual, a mere accidental, concomitant. The ex-

* *Loc. cit.*

† See his *Treatise on Febrile Diseases.*

periments of Mr. Brodie and some other eminent physiologists appear, as far as experiments can prove any thing respecting the more minute actions of the organs of an animal body, to have satisfactorily controverted the notion of increased action of the arteries being an essential circumstance in inflammation. The same observations and experiments also appear to disprove the existence of obstruction to the course of the blood through the capillaries; an opinion which has been successively adopted by Asclepiades, Böerhaave, and Cullen, but which is not supported by any well-ascertained facts.

But how satisfactorily are the queries of Dr. Philip replied to, and how well are the principal phenomena of inflammation explained, by the hypothesis, we would almost say theory, of Bordeu.

"Perhaps what has been termed a congestion of blood," says Bordeu, "and which has been regarded as the cause of inflammation, is only an effect of a particular predisposition that has taken place in the part, the nerves of which have acquired a certain and somewhat violent degree of action, and which is, properly speaking, the cause of inflammation.

"If each trunk of the blood-vessels be surrounded with nerves, as we have supposed, and these nerves become irritated, they will propel the blood in greater quantity, and with much more force than ordinary, towards the ramifications of those vessels, as takes place in glands to which the fluids are carried in greater quantity, and with more force, during the action of secretion.

"If we acknowledge that every part has vessels which do not, in the ordinary state, receive all the fluid they are able to contain,—that is to say, that these vessels, more or less dilated, will receive different parts of the blood,—we might suppose that inflammation has its seat in these vessels, which are so contracted in the ordinary state that they receive only lymph, although they have sufficient capacity to become blood-vessels (admitting the expression) on certain occasions; for example, in the state of inflammation.

"These vessels, then, have the property to be both blood-vessels and lympathics; ordinarily they contain only lymph, because they are contracted so as only to admit that fluid. Are the nerves which accompany them irritated in a certain manner? these vessels dilate themselves and alter their position: they, from being tortuous, become more or less straight, *eriguntur;* and this sort of erection, or of active dilatation, occasions the blood to enter them in greater quantity; besides which, it is also driven into them by the influence of the nerves on the trunk of the principal vessel of the part.

"This is very different from what has been commonly advanced on this point: the tumor has been regarded as the effect of the rush of blood; and, perhaps, indeed, the tumor or congestion *(disposition bouffie)* of the part may occasion the blood to flow to it in greater quantity, and with greater force."

After some observations tending to show the probability of what he has here advanced, Bordeu thus continues:—

"Can we understand the nature of this phenomenon without considering that that which occasions the tumefaction is principally the *erethism* or the particular tension of the nerves and vessels of the part?

"This is not the place to extend this theory:* it is sufficient to remark that an inflamed part is, in a manner, a distinct body *(corps à part)*; at least, it is so for a certain time. It has a sort of action superadded to that which constitutes life; it makes a distinct circle; and what passes in this part resembles what takes place in glands and other organs to which the blood is directed, and where there are species of *torrents* that practitioners have called *raptus*.

"We might, in speaking of this *raptus*, still prove what we have advanced on the subject of the *particular action* of a part, and show how little attention has commonly been paid to this sort of phenomenon by physicians, which they do not know how to explain, any more than they do what regards certain derivations and revulsions, and evacuations of fluids by particular organs, by following what the scholastics say respecting the circulation; but all that would lead us from our principal subject."†

The *erethism* of which Bordeu here speaks is, in an inferior degree, an ordinary function of the animal economy, and is evinced in numerous physiological phenomena: as, the act of blushing; the erection of the papillæ of a woman's breast, on the touch of the lips of her infant; *virgæ erectione vi imaginationis;* the accident that happened to Horace in his way to Brundusium—

"———somnus tamen aufert
Intentum veneri: tum immundo somnia visu
Nocturnam vestem maculant."

---

* Bordeu was, unfortunately for the progress of science at that period, too much inclined to advance some of his finest ideas in an adventitious manner, and then to turn suddenly from them, on all occasions, as he has done on this.

† *Œuvres de Bordeu; Recherches Anatomiques sur la Position des Glandes, et sur leur Action*, § cxxix.

Analogous phenomena are also apparent in the ducts of the secretory glands: as the sudden flow of milk from a woman's breast at the sight of her infant, from which she has been absent a little time; the common act of milking a cow or other animal; (we may here observe, that the explanation given of the mode in which an infant sucks,—that is, by forming a vacuum round the nipple, is obviously insufficient;) and it may be demonstrated by an easy experiment:—stand before a mirror after having fasted a little while; raise the tongue, and think on some favourite species of food; a jet of saliva will instantly spring from the orifices of the glands on each side of the frænum of the tongue. We should, however, turn to Bordeu for the most striking and beautiful illustrations of this phenomenon; but, then, to do it effectually, would require us to transcribe the greater part of his Recherches sur les Glandes.

Mr. John Hunter, it should be remarked, supposed that a sort of active dilatation of the capillaries took place in inflammation; but, without being suspected of want of due respect for the opinions of our great physiologist, we may say that his reasoning on this subject is extremely vague; and, as the name of Bordeu is hardly known in this country, we should observe that he flourished a short time previously to Hunter: the opinions we have here adduced were published in the year 1752.

We must now, though reluctantly, quit Bordeu; but we intreat the younger part of our readers to *study* his works: this, however, they cannot fail being induced to do ere long, for his glory is now just breaking through the clouds that have long obscured it, and the truth of several of his original and beautiful doctrines is daily illustrated by the observations and reflections of the most eminent physiologists. Bordeu was one of the few cultivators of medical science, who, like a Galileo, a Roger Bacon, and an Epicurus, in physics, sprung so far before their contemporaries that they have been lost until men of subsequent generations have arrived to where they terminated their career; and then, and then only, has their merit appeared.

We are disposed, however, before we resign this subject, to adduce a short extract from Bichat, which expresses, in another way (which may, perhaps, be more distinctly intelligible to some persons), what has just been advanced respecting inflammation.

"Is a part irritated in any way, its sensibility immediately alters—it increases. The capillary system, hitherto a stranger to blood, assumes a relation to it; *it calls the blood*, in a manner; the blood flows to it, and remains accumulated in the part until its organic sensibility returns to the ordinary state.

"The penetration of the capillary system by the blood is, then, a secondary effect in inflammation. The principal phenomenon, that which causes all the rest, is the local irritation, which has changed the organic sensibility of the part."*

The arguments of Dr. Grattan in favour of the use of digitalis and mercury in inflammation, are deduced from the ideas and opinions he has formed respecting the nature of that affection; they are now to become the subject of our remarks: we must, however, previously observe, that the use of those remedies for the same purpose is not novel in itself; although really original with respect to Dr. Grattan, since he was led to it by his own particular theoretical views.

Dr. Mossman was, we believe, the first who argued for the utility of digitalis in inflammatory affections, from its influence on the minute arteries: his reflections on this subject were published in the fourth volume of our Journal.

Dr. Farre also, some years since, indicated the mode in which mercury relieves inflammation, on principles similar to those advanced by Dr. Grattan. Mercury had been previously employed in various inflammatory affections by numerous practitioners; but this appears to have been done from observation of the results from its use, rather than from theoretical principles. Even Dr. Rush, who employed mercury in the early stages of phthisis with remarkable success, seems to have considered that its beneficial effects in that disease arose solely from the counter-irritation it produced in the salivary glands; since his constant object was the excitement of salivation.†

We consider that Dr. Grattan has not formed a strictly correct opinion respecting the mode in which digitalis acts on the animal economy: he states that it immediately lowers the action of the heart, and diminishes the frequency of the pulse. This is contrary to the observations of Dr. Saunders and every other physician of eminence who has carefully watched its effects; all of whom concur in asserting, that, when the action of the heart and the frequency of the pulse are below a certain point, (about 130 or 140 in a minute,) it universally increases their force and frequency before it diminishes them. When the pulsations of the arteries are above 150 or 160 in a minute, digitalis has been frequently

---

* *Anatomie générale, Systèmes Capillaires,* § vi.

† See *London Medical and Physical Journal,* vol. xix. p. 47, et passim.

found to lessen them immediately; and so would wine or alcohol under the same circumstances; but it would not thence be proper to call them sedatives even on these occasions.

The state of health is that in which the influence of medicinal substances on the animal economy can be most accurately determined; and, from having this view of the subject, the writer of this critique instituted a series of experiments on himself, with several of what are commonly termed the vegetable poisons. Those with digitalis were repeated several times, under various circumstances, with different objects: this substance constantly increased the force and frequency of the action of the heart and arteries before it diminished them; the lessened degree of action appeared to ensue in consequence of the nervous sensibility becoming depressed from its previous excitement; and, by repeating the dose of the vegetable at short intervals, and constantly and progressively increasing it, preternatural force and frequency of the action of the heart and arteries were maintained during eighteen days, when the experiment was terminated, in consequence of the serious derangement of the bodily and intellectual faculties that was produced. In another experiment, in which the quantity of the vegetable taken was less, and the intervals between the doses longer, the action of the heart and arteries, after having been increased in force and frequency during two days, fell below the natural standard; but even then it was again excited for a short time after each dose, although it did not rise to the ordinary degree on these occasions.

Dr. Grattan also considers that digitalis, whilst it lessens the action of the arteries, increases that of the absorbent vessels; and this appears to be a common opinion: but we have been led to conclude that it has but little influence on the absorbents,—that is, but very little when compared with what it has on the minute ramifications of the arteries. It appears that it is from the continued use of digitalis lessening in a considerable degree the action of the latter, whilst it has but little effect on the former, thus destroying the ordinary balance between exhalation and absorption, that the opinion above stated has arisen.

Dr. Grattan, however, proposes as a question—

"May not digitalis, at the same time that it increases the action of the lymphatics, which every where accompany the capillaries, also stimulate the latter to more forcible contraction, so as to propel their contents, and relieve themselves from morbid distension? Its action on the heart is sedative, as is also its effect on the arterial branches which terminate in the capillaries; but the capillaries evidently possess a structure different from the arteries from which they derive their origin; and hence it is not unphilosophical to suppose that they may be differently affected even by the same medicine."

And he continues to remark—

"Should this opinion be correct, the local action of digitalis on the vessels which are the immediate seat of inflammation will bear a strong analogy to the effects of topical blood-letting; while its sedative effects on the heart and arteries equally resemble those of blood-letting largely practised from the general system. At all events, be this as it may, the increased action of the lymphatics, together with the diminished action of the heart and arteries, are in themselves sufficient to explain whence it is that digitalis is a remedy of such utility in the treatment of inflammation."

We refer our readers to the work of Dr. Saunders on this subject, and to the numerous Memoirs by Drs. Mossman, M'Lean, Drake, Kinglake, Hamilton, Fowler, Penkevil, and several other eminent physicians, which were published in the early volumes of our Journal, for the means of determining this question, which is not a point of mere idle speculation; since, if it be proved that the immediate influence of digitalis on the heart and arteries is that of a stimulant, much caution and reserve are shown to be necessary in its use during the more intense forms of inflammation, and when the action of the arteries is much increased in force; whilst, if it be a direct sedative, no such reserve is necessary. The writers to whom we have referred, concur in stating that it should not be used before the violence of inflammatory action has somewhat subsided. We should, however, mention that Dr. Grattan has premised the use of "moderate venæsection."

"Independently of the power which digitalis evidently possesses of diminishing the *vis a tergo*, (says Dr. Grattan,) it has the further effect of increasing absorption, from the various textures which are supplied with blood-vessels and capillaries. Thus, in this way, it also tends to subdue inflammation; for, as absorption is promoted, the contents of the capillaries must be proportionably diminished, and their turgescence relieved.

"Mercury, like digitalis, acts powerfully on the absorbent system. Although it has a strong tendency to equalize the circulation and to remove congestion, yet it does not diminish the action of the heart or arteries. Its influence is principally exerted on the capillary system, and particularly on that of the absorbents, which it stimulates more effectually and permanently than any other medicine. That it acts immediately on the capillaries, I think, can hardly be disputed, when we consider its well-known effects in changing the nature of various secretions; causing some to become healthy that were before diseased, and altering for the worse others that were healthy previously to its employment. From what has been already said on the subject of digitalis, and the mode in which it probably acts, it would seem that mercury is, in certain cases of disease, even more likely to be of use."

In inflammation of the mucous membrane of the lungs in weak and debilitated constitutions, accompanied with quick laborious breathing, orthopnœa, tumid countenance, and dark colour of the cheeks and lips, the use of digitalis and mercury, with auxiliary remedies,—"such as blisters, expectorants, &c. and sometimes stimulants, when the general debility and absence of acute inflammation appeared to indicate them,"—has been found so beneficial by Dr. Grattan, that "he never lost a patient under those circumstances, when the system was once fairly under the influence of mercury:" and these remarks, he states, apply to inflammation of every other organ as well as the lungs.

*(To be continued.)*

---

*A Treatise on Midwifery; developing new Principles, which tend materially to lessen the Sufferings of the Patient, and shorten the Duration of Labour;* by John Power, Accoucheur, &c. Member of the Royal Medical Society of Edinburgh. pp. 270.—London, T. and G. Underwood. 1819.

*(Concluded from p. 336.)*

"The metastatic action may be determined to various parts of the bodily system; these may be classed under two heads:

"1. The metastatic action may actuate the muscular system.

"2. The metastatic action may produce increased actions of the arterial system.

In entering upon the consideration of metastatic action determined to muscular parts distinct from the uterine system, the author proceeds to enquire more precisely into the nature of that action of the uterus commonly denominated "labour pain." In this investigation, he satisfactorily exposes the inaccurate and contradictory notions of former writers on this subject, and deduces from the facts as they have appeared to himself,—

"1. That the uterine action is not necessarily accompanied by a spasmodic state of the uterine muscles, and consequent pain.

"2. That the most distressing pains of parturition may be totally unconnected with the action of the uterus.

"3. That such pains are inefficacious and unprofitable."

The pains of parturition depend upon two different principles.

"1. The sensations excited in the uterine system itself by the direct action of the parturient energy.

"2. The sensations excited in parts distinct from the uterine system, by the metastasis of the parturient energy from that system to different parts."

The painful sensations from uterine action are either from spasmodic action of the muscular structure of the uterus, or from pressure produced by the contractions of the womb upon the os uteri, vagina, and other parts connected with the passage for the child.

The pains arising from metastasis of the parturient energy to other muscular parts are, in general, of a very distressing nature, and are usually described as "cramping, cutting, grinding, rending." When the rectum or bladder become the seat of metastatic action, a sensation of the expulsive actions of those organs is experienced, and a sense of bearing down, which is often confounded with the regular propellent action of the uterus on its contents. Other peculiarities of these pains are observable, and their independence of true contractile effort of the uterus is demonstrable by the flaccid feel of this organ to the hand imposed upon the abdomen during the paroxysm. With these views of the author, we feel disposed very fully to coincide; and we think it would be difficult to withhold conviction from his very luminous exposition of them.

On the metastatic excitement of the arterial system, according to Mr. Power's opinions, depend several adventitious phenomena of parturition, which we are inclined to consider as not having before been placed in so intelligible a scheme of relationship. The excitement may be more or less general, or restricted to an individual part of the system, presenting appearances modified by the peculiarities of its several seats of action. The author is of opinion that, amongst other effects, the metastatic action may excite determination to the brain, occasioning

"*a.* A state of convulsion known as puerperal convulsion.
"*b.* A state of syncope or hysteria.
"*c.* It may give rise to febrile state."

These are subjects of the highest interest, and will command attention to any plausible hypothesis which promises to throw light on their hitherto obscure nature.

For the suspension of the parturient energy, Mr. Power assigns the following causes:—

"1. The nervous power may be insufficiently produced.
"2. The nervous power may have been exhausted.
"3. The irritations of the uterine orifice, which should determine the nervous power to actuate the uterine muscles, may be insufficient to excite such effect."

These causes are all separately considered, and made to appear adequate to the production of the effect assigned to them.

On the deviations from mechanical obstruction to the expulsion of the uterine contents, the author touches very slightly, giving merely the outlines of a very perspicuous arrangement of them.

The deviations from accidental circumstances are enumerated under the following heads:—A. Premature expulsion. B. Hæmorrhage. C. Retention of the placenta. D. Rupture of the uterus. E. Laceration of the perinæum, &c. F. Inversion of the uterus. G. Extra-uterine conceptions. H. Plurality of children. I. Protrusion of the umbilical cord.

The consideration of these several circumstances evinces the same clearness of conception, accuracy of judgment, and habitual attention to order, which mark every page of this masterly production. We should exceed our limits were we to indulge our inclination to follow the author minutely through every section.

In the fourth chapter Mr. Power presents us with a synoptical arrangement of the varieties of the parturient state, founded on the principles which he has in the former chapters so luminously developed. His distribution is lucid, natural, and complete.

With this synopsis the author closes the first part of his book.

The second part of Mr. Power's work comprises practical observations relative to parturition; and, relying on the correctness of the principles which he has endeavoured to establish, he infers, and justly we think, that their influence in practice must be considerably important. His work is professedly but a partial treatise on Midwifery, either as a science or as an art; and he, therefore, in his practical considerations, limits himself to those points which seem to be immediately dependant on his own peculiar principles.

The progress of a perfectly natural labour calls for little or no interference from art; and, consequently, the indications are rather to guard against aberrations, than to offer active assistance. The general view which the author here takes of this class of parturition is clear and correct, and his practical inferences highly judicious.

In the obstetric department of the profession, as well as in the other branches, the attention of the practitioner is mainly directed to deviations from a state of natural and healthy action. The class dystocia, therefore, is that which becomes chiefly interesting in the view of the practical reader; and of this class the second part of the work under notice treats principally of the first order, under Mr. Power's arrangement, or "Unnatural Parturition, arising from derangement of the parturient energy."

The most commonly occurring genus of this order is that of "labour with painful uterine action." If simply confined to this character, it rarely calls for interference; and the author refers the accustomed treatment to the respective cases, rather to show the inutility than to enforce its adoption. At the same time, circumstances are pointed out which indicate the advantageous interference of art.

"Labour with partial uterine action" is not of frequent occurrence, and is so nearly connected with the subsequent genus in its causes, effects, and treatment, as scarcely to demand a separate notice.

In Mr. Power's view, the most frequent cases of protracted parturition, unconnected with mechanical impediment, will be found to belong to the genus.

"Labour with metastatic determination to muscular parts." In the history of the symptoms, the distinction of specific varieties, the causes, the prognosis and diagnosis, the author enters into a detail highly interesting to the practical reader, and marked by this writer's usual clearness of conception and methodical arrangement; but we should be trespassing too much on the forbearance of the general reader of our pages were we to dilate too far on a subject remote from the pursuits of a numerous body of the profession.

On the treatment of these cases we shall not go into the minute consideration which the author bestows upon it, but shall briefly present to our readers the novel feature of Mr. Power's practice, upon which he himself looks with a kind of parental attachment, and to which he attributes an efficacy that we have no reason to anticipate will not be confirmed by experience. He says—

"Although friction of the abdomen has been recommended as an adjuvant in producing a more early and proper expulsion of the placenta, the author is not aware that it has, in any instance, been used or treated of for the relief of protracted parturition; he hopes, therefore, that he may, without presumption, assert a claim to originality in proposing its introduction for such intention.

"On first entering upon the practical duties of midwifery, it became obvious to him that the effects of parturition were by no means proportionate to what were considered its efforts, and that the pain, which was to be regarded as the measure of those efforts, bore no relation to the degree of progress; that, in many instances, a comparatively slight degree of pain would, at one time, produce a rapid advancement of the labour; whereas, at other times, and even in the same individual case, under a series of most severe and unspeakable sufferings, little or no advancement would be made. Although aware that the observation was not new, the facts impressed him strongly; and, as he could recollect no explanation of the inconsistency in the various lectures he had attended, or writings he had studied, the subject was made an object of his serious consideration. He soon became convinced that the unprofitable pains, above noticed, were truly and *ipso facto* extra-uterine; that they produced no effect on the os uteri or expulsion of the child; and that they consisted of spasmodic affections of the surrounding parts.

"The obvious inference which now presented itself was, that their removal ought to be attempted upon the principle of relieving spasm. Having been long in the habit of employing vigorous friction for the removal of affections of the latter kind, he was naturally induced to extend its use to answer this new indication; the result exceeded his most sanguine expectation,—protracted cases, and that dread of meeting with them which had been implanted in his mind by the expectation that their occurrence would constitute the most disagreeable and perplexing part of his professional labours, vanished under its use; and he has since continued its employment, with the most happy effects, in a large proportion of the cases which have come under his care."

With the admission that some cases failed to be relieved by this practice, experience has confirmed the author's first expectations that in a majority of cases the relief afforded would be decided and ample. Mr. Power proceeds to speculate with much ingenuity on the *modus agendi* of this remedial power, and to impart instruction on the cases where it is admissible, and the manner of applying it. All this will be read with interest by the obstetric practitioner; and we think that, uninstructed by experience, he will in general be disposed to coincide with the views here exposed. He concludes his observations on this subject in the following passage:—

"By a full and careful attention to the rules laid down, the author's experience leads him to assert the possibility of terminating happily, in a comparatively short period, almost every case of protracted parturition, which can fairly be referred to the present genus."

After treating briefly of the means of producing suspension of the parturient action where this may be deemed advisable, he dismisses the consideration of this genus of labour thus—

"If, after every attempt, our intentions should be frustrated, and the desired termination alarmingly protracted, instrumental aid must be called in: of this kind of assistance the forceps can only become necessary, and the system will never be found to have sustained so much injury as to render the use of these desirable before the parts are sufficiently dilated to make their application admissible.

"The above may be thought to encourage instrumental interference; but, in an active practice of twelve years, the author has not found it necessary to apply the forceps a dozen times, and these were cases truly referrable to other deviations. On the contrary, he has been successful in a number of protracted cases under his peculiar treatment, which he believes would otherwise have required such interference."

Another genus under this order, in Mr. Power's arrangement, comes next to be considered:—"Labour with metastatic determination to the arterial system." An admission, however, of the hypothesis involved in the definition of this genus cannot be made but upon strong grounds, and an extended view of correlative facts. A series of accurate observations are wanting to give sanction to its establishment; yet we confess that the phenomena associated with the assigned cause bear an apparent affinity to it, which we do not feel much inclined to call in question. We think the suggestion of the author entitled to the deliberate attention of all those who feel an interest in the development of pathological causes. The present genus, admitting the author's system, embraces morbid conditions of the parturient process, of the highest importance both to the patient and practitioner; and courts every view which promises an accession to the intelligence of its connected causes. Labour with convulsions, with syncope or hysteria, or accompanied by fever, is always too alarming not to engage warmly the feelings of every party interested. The author enters upon the investigation of these several species with diffidence, and treats them with a brevity which does not spring from their unimportance, but from a want of confidence in his own power of illustrating them. We trust that one so eminently qualified, by accuracy of observation, by correctness of inductive faculty, and by solidity of judgment, will not fail hereafter to elucidate these as well as other parts of his subject, which he has confessedly, in the present essay, left insufficiently examined.

Three other less important genera, connected with deficient parturient energy, are practically considered; and an appendix of illustrative cases closes the volume. Were the works which issue from the medical press all written with the same legitimate view, the same candour, and the same talent, the office of a reviewer would shortly become obsolete.

---

*Observations on the Prevalence of Fever, in various Parts of the United Kingdom; and on the eminent Utility of Houses of Recovery: exhibiting the great Advantages that would result from such an Institution for the Reception of the Sick Poor of Bristol and Clifton.* By D. J. H. Dickson, M.D. F.R.S. Ed. and L.S.; Fellow of the Royal College of Physicians of Edinburgh; Physician of the Fleet; one of the Physicians to the Clifton Dispensary, &c.—8vo. pp. 34. 1819.

We take up this pamphlet for the purpose of pointing it out as deserving of the attention of our readers, rather than from an intention to enter into a regular consideration of its contents; for, although it is chiefly written for local purposes, the arguments advanced in it are applicable to every large town in the kingdom. We know it has been observed, that most of them already contain hospitals or infirmaries, which are open for the reception of fever-patients. Such a remark, it is evident, can only come from those who continue to doubt of the contagious properties of the fever that has of late been prevalent to such a serious extent; but, even in this view, it shows an imperfect consideration of the subject. The state of patients with typhous fever; the attentions and treatment they require; the necessity of surrounding quietness, &c. are circumstances which render a general infirmary improper for their reception.

Dr. Dickson has adduced in this pamphlet a view of the present state and late progress of the prevalent fever in the chief parts of the kingdom, and a condensed account of the opinions of the most eminent and experienced physicians respecting its character, and the mode in which it is propagated. The facts thus adduced lead to some forcible arguments in favour of the measure here proposed; and we hope that the laudable zeal evinced by the author, and the judicious reflections he has advanced, will excite similar sentiments in the minds of the public, and convince them that, in effecting the proposed object, they will not only act in conformity with the most urgent dictates of charity and benevolence, but also contribute in the most effectual manner to the preservation of their own welfare.

*Remarks on the Causes, Prevention, and Management, of the present prevailing Epidemic, commonly called Typhous Fever; for the use and benefit of the People.* By W. O. Porter, M.D. one of the Physicians to the Bristol Dispensary, &c. &c.—8vo. pp. 53. 1819.

To those who were previously unacquainted with the character of the author, we think that the title of this pamphlet might convey an incorrect idea of the nature and object of its contents. It is not written with the vain, and commonly self-interested, intention to persuade the public that they will, by its assistance, be enabled to treat and cure typhous fever; but to show them the nature of that disease, the mode in which it is propagated, the appropriate prophylactic measures, to combat existing prejudices respecting the necessity of wine, &c. in fevers attended with debility; and, finally, to give persons in general some idea of the appropriate mode of treatment, and teach them the necessity of strictly and zealously conforming to the advice of a judicious medical attendant. We have perused it with great pleasure, and think that the benefits it is calculated to diffuse are very considerable. It is an excellent work to put into the hands of the refractory attendants of fever-patients; and medical practitioners will do well in providing themselves with a few copies of it for such a purpose: it might have such an influence on their minds as the coincidence of the opinion of another physician is commonly seen to produce. The style of it is just that in which a work, having such an object, should be written: it has a little of the honey which, Montaigne says, the good physician places on the edge of the cup containing the potion that is to preserve the life of the wayward child. It is, at the same time, not without observations and reflections that merit the attention of many members of the profession, who may be instructed, as well as amused, by Dr. Porter's reflections on contagion.

